# Predictive factors for major postoperative complications related to gastric conduit reconstruction in thoracoscopic esophagectomy for esophageal cancer: a case control study

**DOI:** 10.1186/s12893-018-0348-9

**Published:** 2018-03-06

**Authors:** Shinichiro Kobayashi, Kengo Kanetaka, Yasuhiro Nagata, Masahiko Nakayama, Ryo Matsumoto, Mitsuhisa Takatsuki, Susumu Eguchi

**Affiliations:** 1 Department of Surgery, Nagasaki University Graduate School of Biomedical Sciences, Sakamoto 1-7-1, Nagasaki, 8528102 Japan; 2 Center for Comprehensive Community Care Education, Nagasaki University Graduate School of Biomedical Sciences, Sakamoto 1-12-4, Nagasaki, Japan

**Keywords:** Esophageal cancer, Thoracoscopic surgery, Esophagectomy

## Abstract

**Background:**

Regardless of developments in thoracoscopic esophagectomy (TE), postoperative complications relative to gastric conduit reconstruction are common after esophagectomy. The aim of the present study was to evaluate the predictive factors of major complications related to gastric conduit after TE.

**Methods:**

From 2006 to 2015, 75 patients with esophageal cancer who underwent TE were evaluated to explore the predictive factors of major postoperative complications related to gastric conduit.

**Results:**

Patients with major complications related to gastric conduit had a significantly longer postoperative hospital stay than patients without these complications (*P* <  0.01). Multivariate analysis demonstrated that three-field lymph node dissection (3FLND) and high serum levels of creatine phosphokinase (CPK) and C-reactive protein (CRP) at 1 postoperative day (1POD) after TE were significant predictive factors of major complications related to gastric conduit [odds ratio (OR) 5.37, 95% confidence interval (CI) 1.41–24.33, *P* = 0.02; OR 5.40, 95% CI 1.60–20.20, *P* <  0.01; OR 5.07, 95% CI 1.47–20.25, *P* = 0.01, respectively]. The incidence rates of major complications related to gastric conduit for 0, 1, 2, and 3 predictive factors were 5.3%, 18.8%, 58.8%, and 85.7%, respectively (*P* <  0.01).

**Conclusions:**

Two or more factors in 3FLND and the high levels of CPK and CRP at 1POD after TE were identified as the risk model for major complications related to gastric conduit after TE.

**Trial registration:**

UMIN Clinical Trials Registry, ID: UMIN000024436, Registered date: Oct/17/2016.

## Background

Although esophagectomy remains the curative treatment for patients with esophageal cancer, this procedure is accompanied by high incidences of complications [1, 2]. The rates of morbidity and mortality after esophagectomy in large national databases were reported to be from 42% to 50% and 2.85% to 4.3%, respectively [3–7]. Recent developments and improvements in thoracoscopic esophagectomy (TE) have reduced severe pulmonary complications after esophagectomy [8]. However, postoperative complications related to gastric conduit reconstruction are still common after esophagectomy [9]. Regarding cervical anastomotic complications after esophagectomy, leak and stricture formation are major issues [10, 11]. In particular, ischemia of the proximal portion of the graft predisposes these patients to a high incidence of anastomotic complications after esophagectomy [12]. Less commonly, severe graft ischemia can lead to transmural necrosis. Thus, early diagnosis of an ischemic reaction may facilitate appropriate postoperative management and therapeutic intervention to prevent leakage, strictures and necrosis. The aim of the present study was to determine the predictive factors of severe gastric conduit-related postoperative complications.

## Methods

### Patient population and operations

From 2006 to 2015, 105 patients with esophageal cancer underwent esophagectomy and lymph node dissection at the Department of Surgery at Nagasaki University Hospital. Treatment plans for each patient were provided according to the clinical guidelines edited by the Japan Esophageal Society [13]. We chose open esophagectomy for the patients with severe adhesions in the chest or invasive neoplasia with lymph node involvement. Thirty patients were excluded because they required open esophagectomy with lymph node dissection. Seventy-five consecutive patients with esophageal cancer who underwent TE were retrospectively studied to evaluate the predictive factors for major complications related to gastric conduit after TE. The rules for classification and staging corresponded to the 7th edition of the International Union Against Cancer (UICC)/American Joint Committee on Cancer (AJCC) Tumor Node Metastasis (TNM) staging system [14].

TE was performed from the right side in the left lateral position. Esophagectomy with lymphadenectomy in the mediastinum and around both recurrent nerves were performed. In the abdominal section, hand-assisted laparoscopic gastrectomy was performed to remove the mobilized esophagus with lymphadenectomy around the left gastric artery and aorta. After mobilization of the full stomach and esophagus, a gastric conduit was created by dividing the lesser curve of the stomach. The right gastric and right gastroepiploic artery provided the vascular supply to the created gastric conduit. In 73 patients, the gastric conduit was pulled up in the post-sternal route; in 2 patients, it was pulled up in the post-mediastinal route. The esophagogastrostomy was performed in the neck by end-to-side anastomosis. A 21-mm or 25-mm intraluminal stapler was used as the stapling device (CDH21, CDH25, Ethicon Ltd., Edinburgh, United Kingdom). The inserted part of the gastric conduit was crossed by linear stapling. All staple lines were oversewn.

Three-field lymph node dissection (3FLND) was performed in patients who had upper thoracic esophageal cancer or middle or lower thoracic esophageal cancer with lymph node metastasis in the neck region or around the right recurrent nerve [15].

This study was approved by the Ethics Committee of Nagasaki University Hospital (16082215). The written informed consent from the patients was waved from the Ethics Committee because the information on the opportunity to opt out was presented on the web site (http://www.mh.nagasaki-u.ac.jp/research/rinsho/patients/open_surgery2.html). This study was registered in the UMIN Clinical Trials Registry as UMIN000024436.

### Postoperative management

The nasogastric tube was removed before anastomosis. On the first postoperative day (1POD), transintestinal nutrition was started from a jejunostomy feeding tube to prevent postoperative complications [16]. In the first three postoperative days, the patients without hoarseness and aspiration pneumonia started to drink fluids, followed by a soft diet.

### Definition of major postoperative complications related to gastric conduit reconstruction

Major complications related to gastric conduit after TE were defined as anastomotic leakage, refractory anastomotic strictures, and gastric conduit necrosis. Anastomotic leakage was defined as fistula formation that required any invasive treatment (Clavien-Dindo classification of grade III or more). Anastomotic strictures were defined as the presence of a lumen requiring endoscopic balloon dilatation for the passage of a normal endoscope (9.2 mm diameter) with symptomatic dysphagia. Refractory esophageal strictures were defined as more than 5 sessions of balloon dilation 6 months after the operation [17, 18]. Gastric conduit necrosis was defined as a severe ischemic condition that required resection of the gastric graft.

### Statistical analysis

The data are expressed as the means ± standard deviation (SD) or medians and interquartile ranges (IQR). The relationships among major complications related to gastric conduit and age, body mass index (BMI), total operation time, operation time of thoracic surgery, and C-reactive protein (CRP) at 1POD were evaluated using Student’s t-tests. The relationships between major postoperative complications related to gastric conduit reconstruction and other values were evaluated using Wilcoxon’s tests. The relationships among anastomotic leakage and age, body mass index (BMI), total operation time, operation time of thoracic surgery, and C-reactive protein (CRP) at 1POD were evaluated using Student’s t-tests. The relationships between anastomotic leakage and other values were evaluated using Wilcoxon’s tests. The relationships among refractory anastomotic strictures and age, body mass index (BMI), total operation time, operation time of thoracic surgery, and C-reactive protein (CRP) at 1POD were evaluated using Student’s t-tests. The relationships between refractory anastomotic strictures and other values were evaluated using Wilcoxon’s tests. Receiver operating characteristic (ROC) curves and the area under the ROC curve (AUC) were used to assess the feasibility of using CRP and creatine phosphokinase (CPK) at 1POD as diagnostic tools for major complications related to gastric conduit [19]. The 95% CI values greater than 0.5 for AUC indicated that prediction was better than chance [20]. The patients were divided into two groups according to the cut-off values of CRP and CPK at 1POD. The relationships of categorical clinical factors between the groups were analyzed using chi-square tests or Fisher’s exact tests. A Fisher’s exact test was applied if the theoretical frequency was less than five. Probability values (P) less than 0.05 were considered statistically significant. Multiple logistic regression (stepwise) models were developed, and odds ratios (OR) were used to evaluate predictive factors associated with major complications related to gastric conduit. The Cochrane-Armitage trend test was used to test for a linear trend in the proportion of patients who developed major postoperative complications related to gastric conduit reconstruction according to numbers of predictive factors. All statistical analyses were performed using SAS-JMP programs for Windows (SAS Institute Inc., Cary, NC).

## Results

### Patient characteristics

The clinical characteristics of the 75 patients, which included 18 females and 57 males, are summarized in Table 1. The average age of all patients was 61.3 ± 8.1 years. The average BMI of all patients was 21.3 ± 2.7. Preoperative chemotherapy was performed in 51 patients (68.0%). Three patients (4.0%) were diagnosed with adenocarcinoma, and 72 patients (96.0%) were diagnosed with squamous cell carcinoma. According to the TNM classification, 47 patients (62.7%) had tumors more advanced than stage I. 3FLND was performed in 23 patients (30.7%). The average operating time was 605 ± 114 min. The median estimated blood loss was 370 g (IQR 270–600). Blood transfusion was performed in 7 patients (9.3%). The median length of the postoperative hospital stay was 27 days (IQR 20–39).

**Table 1:** Patients’ characteristics

Characteristic	Values
Age (year)	61.3 ± 8.1
Gender (Male, Female)	57, 18
BMI	21.3 ± 2.7
Preoperative chemotherapy	51 (68.0%)
TNM Stage (I, II(IIA, IIB), III(IIIA, IIIB, IIIC), IV)	28, 19 (7, 12), 24 (13, 7, 4), 4
Total operating time (min)	605 ± 114
Operation time of thoracic surgery (min)	331 ± 73
Blood loss (g)	370 (270–600)
Blood transfusions	7 (9.3%)
3-field lymph node dissection	23 (30.7%)
Paroxysmal atrial fibrillation	13 (17.3%)
Vasopressor agents	8 (10.7%)
WBC (10^3/μl) at 1POD	9.4 (7.7–12.3)
CRP (10^4 μg/L) at 1POD	9.2 ± 2.4
Lactic acid (mmol/L) at 1POD	1.8 ± 1.2
CPK (IU/L) at 1POD	961 (670–1504)
Postoperative hospital stay (days)	27 (20, 39)

### Major complications related to gastric conduit reconstruction after TE

**Fig. 1:**
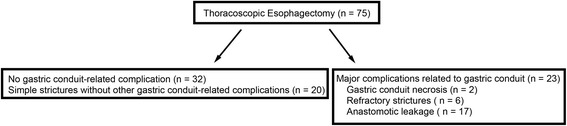
Major complications related to gastric conduit reconstruction in 75 patients who underwent thoracoscopic esophagectomy. Seventeen patients developed anastomotic leakage. Refractory esophageal strictures were defined as more than 5 sessions of balloon dilation 6 months after the operation. Six patients developed refractory esophageal strictures. Two patients who had developed anastomotic leakage developed refractory esophageal strictures

The major complications related to gastric conduit after TE are summarized in Fig. 1. Twenty-three patients (30.7%) developed major complications related to gastric conduit reconstruction after TE. Anastomotic leakage occurred in 17 patients who required drainage to manage infectious conditions. No patients died within 30 days after the operation due to anastomotic leakage. A stricture occurred in 33 patients who required endoscopic balloon dilation. Twenty patients developed simple esophageal strictures without other gastric conduit-related complications. Seven patients developed anastomotic leakage followed by simple esophageal strictures. Six patients developed refractory esophageal strictures. All patient with refractory strictures developed symptomatic strictures within 2 months after TE (28.0 ± 7.0 days). Two patients developed anastomotic leakage followed by refractory esophageal strictures. Two patients had gastric conduit necrosis, and one of these two patients died due to non-occlusive mesenteric ischemia after resection of the necrotic gastric conduit. The length of postoperative hospital stay after TE in the patients with major complications related to gastric conduit (39 days, IQR 28–47) was significantly longer than in those without these complications (22 days, IQR 19–28) (*P* <  0.01).

### Predictive factors for the development of major complications related to gastric conduit after TE

**Fig. 2:**
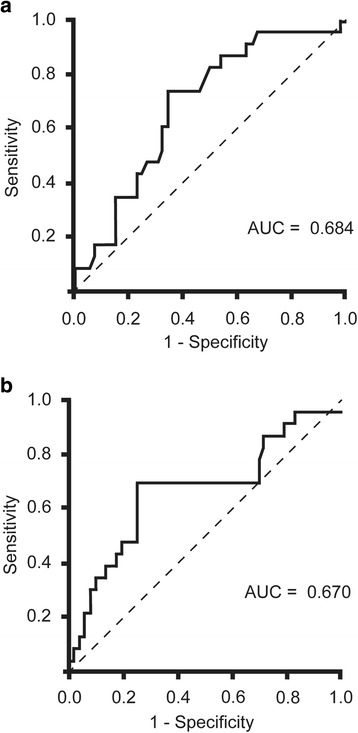
ROC curve analysis of CRP (**a**) and CPK (**b**) at the first postoperative day after thoracoscopic esophagectomy. At a threshold of 9.6 x 10^4^μg/L CRP at 1POD, the optimal sensitivity and specificity were 73.9% and 65.4%, respectively, in patients developing major postoperative complications related to gastric conduit reconstruction. At a threshold of 1164 IU/L CPK at 1POD, the sensitivity and the specificity were 69.6% and 75.0%, respectively, in patients developing major postoperative complications related to gastric conduit reconstruction. ROC, receiver operating characteristic; CRP, C-reactive protein; POD, postoperative days; CPK, creatine phosphokinase. AUC, area under the ROC curve; CI, confidence interval

The predictive factors for developing major complications related to gastric conduit are shown in Table 2. In a univariate analysis, the predictive factors for developing major complications related to gastric conduit included age, 3FLND and levels of CRP and CPK at 1POD. The AUC for CRP and CPK at 1POD was 0.684 (95%CI; 0.546–0.796) and 0.670 (95%CI; 0.514–0.796). ROC curve analysis also identified the following cut-off values for CRP and CPK at 1POD: 9.6 × 10^4 μg/L and 1164 IU/L, respectively (Fig. 2). At a threshold of 9.6 × 10^4 μg/L for CRP at 1POD, the optimal sensitivity and specificity were 73.9% and 65.4%, respectively, in patients developing major complications related to gastric conduit. At a threshold of 1164 IU/L for CPK at 1POD, the sensitivity and the specificity were 69.6% and 75.0%, respectively.

**Table 2:** Univariate analysis for factors predicting major complications related to gastric conduit after TE

	Postoperative complications related to gastric conduit reconstruction		*P*-value
	Negative (*n* = 52)	Positive (*n* = 23)	
Age (years)	62.9 ± 7.2	57.5 ± 8.7	< 0.01
Gender (Male, Female)	37, 15	20, 3	N.S.
BMI	21.4 ± 2.7	21.1 ± 2.7	N.S.
Preoperative chemotherapy	21 (40.4%)	13 (56.5%)	N.S.
TNM Stage (I, II, III, IV)	22, 10, 18, 2	6, 9, 6, 2	N.S.
Total operation time (min)	604 ± 113	606 ± 116	N.S.
Operation time of thoracic surgery (min)	337 ± 77	318 ± 64	N.S.
Blood loss (g)	380 (303–623)	340 (200–500)	N.S.
Blood Transfusion	5 (9.6%)	2 (5.2%)	N.S.
3-field lymph node dissection	12 (23.1%)	11 (47.8%)	0.03
Paroxysmal atrial fibrillation	10 (19.2%)	3 (13.0%)	N.S.
Vasopressor agents	5 (9.6%)	3 (13.0%)	N.S.
WBC (10^3/μl) at 1POD	9.7 (8.2–12.8)	8.9 (7.0–11.5)	N.S.
CRP (10^4 μg/L) at 1POD	8.7 ± 0.3	10.3 ± 0.5	< 0.01
Lactic acid (mmol/L) at 1POD	1.4 (1.1–1,8)	2.1 (1.2–2.7)	N.S.
CPK (IU/L) at 1POD	890 (620–1309)	1277 (675–2041)	0.02
Postoperative hospital stay (days)	22 (19–28)	39 (28–47)	< 0.01

The predictive factors for developing major complications related to gastric conduit are shown in Tables 3 and 4. In a univariate analysis, the predictive factors for developing anastomotic leakage related to gastric conduit included levels of CRP and CPK at 1POD.

**Table 3:** Univariate analysis for factors predicting anastomotic leakage after TE

	Anastomotic leakage		*P*-value
	Negative (*n* = 58)	Positive (*n* = 17)	
Age (years)	62.3 ± 7.8	57.8 ± 8.3	N.S.
Gender (Male, Female)	43, 15	14, 3	N.S.
BMI	21.3 ± 2.7	21.1 ± 2.8	N.S.
Preoperative chemotherapy	25 (43.1%)	9 (52.9%)	N.S.
TNM Stage (I, II, III, IV)	22, 13, 20, 3	6, 6, 4, 1	N.S.
Total operation time (min)	608 ± 112	594 ± 122	N.S.
Operation time of thoracic surgery (min)	338 ± 76	305 ± 60	N.S.
Blood loss (g)	380 (290–608)	350 (235–615)	N.S.
Blood Transfusion	6 (10.3%)	1 (5.9%)	N.S.
3-field lymph node dissection	15 (25.9%)	8 (47.1%)	N.S.
Paroxysmal atrial fibrillation	10 (17.2%)	3 (17.7%)	N.S.
Vasopressor agents	7 (12.1%)	1 (5.9%)	N.S.
WBC (10^3/μl) at 1POD	9.7 (8.2–12.8)	8.9 (7.0–11.5)	N.S.
CRP (10^4 μg/L) at 1POD	8.7 ± 2.4	10.5 ± 2.0	< 0.01
Lactic acid (mmol/L) at 1POD	1.5 (1.1–2.0)	2.0 (0.7–2.6)	N.S.
CPK (IU/L) at 1POD	919.5 (629–1400)	1232 (683–2177)	< 0.05
Postoperative hospital stay (days)	22 (19–28)	42 (30–47)	< 0.01

**Table 4:** Univariate analysis for factors predicting refractory anastomotic strictures after TE

	Refractory anastomotic strictures		*P*-value
	Negative (*n* = 69)	Positive (n = 6)	
Age (years)	61.4 ± 8.2	59.0 ± 6.4	N.S.
Gender (Male, Female)	51, 18	6, 0	N.S.
BMI	21.4 ± 2.7	21.1 ± 2.7	N.S.
Preoperative chemotherapy	39 (56.5%)	2 (33.3%)	N.S.
TNM Stage (I, II, III, IV)	27, 16, 22, 4	1, 4, 1, 0	N.S.
Total operation time (min)	604 ± 113	606 ± 116	N.S.
Operation time of thoracic surgery (min)	331 ± 75	329 ± 55	N.S.
Blood loss (g)	380 (303–623)	340 (200–500)	N.S.
Blood Transfusion	6 (8.7%)	1 (16.7%)	N.S.
3-field lymph node dissection	22 (29.3%)	1 (16.7%)	N.S.
Paroxysmal atrial fibrillation	12 (17.4%)	1 (16.7%)	N.S.
Vasopressor agents	7 (10.1%)	1 (16.7%)	N.S.
WBC (10^3/μl) at 1POD	9.7 (8.2–12.8)	8.9 (7.0–11.5)	N.S.
CRP (10^4 μg/L) at 1POD	9.0 ± 2.3	10.9 ± 3.6	N.S.
Lactic acid (mmol/L) at 1POD	1.4 (1.1–1,8)	2.1 (1.2–2.7)	N.S.
CPK (IU/L) at 1POD	890 (620–1309)	1214 (675–2041)	N.S.
Postoperative hospital stay (days)	20 (20–35)	41 (25–61)	N.S.

**Fig. 3:**
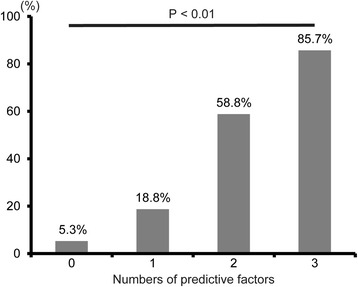
Prevalence of major complications related to gastric conduit reconstruction compared according to the number of predictive factors after thoracoscopic esophagectomy. *P*-value based on the Cochrane-Armitage trend test

When a multiple logistic regression analysis was performed to evaluate confounding factors, 3FLND and the levels of CPK and CRP at 1POD were found to be significantly associated with developing major complications related to gastric conduit (Table 5). The incidence rates of these complications for 0, 1, 2, and 3 predictive factors were 5.3% (1/19), 18.8% (6/32), 58.8% (10/17), and 85.7% (6/7), respectively (Fig. 3). There was a strong trend toward increasing the prevalence of major complications related to gastric conduit based on the number of predictive factors (*P* <  0.01). The accuracy of 2 or more factors for major complications related to gastric conduit after TE was 0.800.

**Table 5:** Multivariate analysis for factors predicting major complications related to gastric conduit after TE

	Odds ratio	*P*-value	95% CI
Age (years)	0.92	0.06	(0.85–1.00)
3-field lymph node dissection	5.37	0.02	(1.41–24.33)
CRP at 1POD (high / low)	5.07	0.01	(1.47–20.25)
CPK at 1POD (high / low)	5.40	< 0.01	(1.60–20.20)

## Discussion

Our results showed that major postoperative complications related to gastric conduit were frequently present after TE and significantly prolonged the length of the postoperative hospital stay. We first evaluated the predictive factors for major complications related to gastric conduit after TE. Our study investigated the number of factors that could predict these complications after TE.

TE has been shown to reduce pulmonary complications and the length of postoperative hospital stay [8, 21, 22]. These results indicate a faster postoperative recovery in patients after TE than in patients after open esophagectomy. However, the morbidity after TE remains high (approximately 30–40%) [1, 2, 23], and preventing gastric conduit-related complications after TE remains difficult. Our data are similar to the Japanese nationwide web-based database and other articles regarding BMI, operation time, the rate of anastomotic leakage, and postoperative days [5, 7, 24, 25]. There is a therapeutic benefit in predicting the postoperative complications of gastric conduit reconstruction after TE. The 3 predictive factors identified in this study may facilitate the decision to delay oral intake and perform early interventional treatments, such as re-operation, drainage and dilation, after TE.

Our study showed that 3FLND led to major complications related to gastric conduit. Lymph node dissection for thoracic esophageal cancer is controversial, and whether 3FLND or 2-field lymph node dissection (2FLND) is better remains a subject of debate [26–29]. The advantages and disadvantages of 3FLND remain controversial when compared to 2FLND of esophagectomy [30, 31]. One meta-analysis showed that 3FLND improves the overall survival rate but leads to more major complications than 2FLND [32, 33]. Anastomotic leakage is likely linked to cervical lymph node dissection due to inflammation and reduced angiogenesis around the anastomotic area [34], which strongly supports our results. However, future studies should determine whether 3FLND or 2FLND is better according to the patient’s physical condition and tumor staging.

The retrosternal route in almost all cases was applied to the gastric conduit of reconstruction after esophagectomy. There are several advantages of this method for the management of local recurrence, including fewer complications in gastric conduit and a short route in the retrosternal route of reconstruction [13, 35, 36]. In RCT studies, both posterior and anterior mediastinal routes of reconstruction were associated with similar surgical outcomes after esophagectomy for cancer [37]. In the Japanese registry, the retrosternal route of reconstruction was selected in 34% of patients, although the posterior mediastinal route was used for reconstruction in 41.3% of patients [38]. Thus, the route of reconstruction remains controversial.

High CRP levels after esophagectomy are reported to precede the clinical diagnosis of postoperative infectious complications [39, 40]. With regard to postoperative infectious complications, there is no difference between patients with and without postoperative infectious complications on 1POD [39, 40]. Consistent with previous reports, our results showed that some infectious complications developed but hardly affected the serum CRP levels on 1POD after TE. Moreover, TE minimizes lung injury and severe pulmonary complications after esophagostomy. Thus, high CRP levels on 1POD may be induced in response to surgical trauma and gastric conduit ischemic conditions after TE. We also identified high CPK levels as a predictive factor for major complications related to gastric conduit after TE. CPK was also reported as a biomarker of ischemic small bowel disease in animal models [41, 42]. CPK may reflect not only ischemic changes in the muscle layer of the gastric conduit but also inflammation around the muscle layers of the neck, as the CPK level is not generally a good biomarker for bowel ischemia [43]. In open esophagectomy, high CPK levels may be observed in patients without major complications related to gastric conduit because of the large incision in the thoracic field.

Postoperative endoscopic examination is a highly accurate method to evaluate reconstruction of the gastric conduit after esophagectomy [12, 44–46]. However, endoscopic examination is complex and invasive after esophagectomy. Thus, these predictive factors after TE are useful to select patients who may benefit from endoscopic examination.

Published results have been inconclusive as to which anastomotic technique is ideal for esophagectomy [9, 47–51]. Thus, surgeons base their choice of anastomotic technique on personal preference. We applied end-to-side anastomosis with an intraluminal stapler in this study. Cervical anastomosis using a stapler more frequently causes anastomotic strictures than other techniques [9, 52]. However, almost all patients show improved anastomotic strictures after three or fewer dilatations within several months [52]. Thus, the ischemic condition of the gastric conduit may influence anastomotic healing in patients who develop refractory strictures [49, 52].

Our study has several limitations. First, our study was performed at a single institution, and further prospective studies are needed at multiple institutions. Second, the accuracy of the predictive factors is somewhat low. Third, almost all patients were diagnosed with squamous cell carcinoma. Thus, transthoracic extended radical esophagectomy with 3-field lymph node dissection is included in our data. The invasive procedure caused delayed recovery of the patients and resulted in a relatively long postoperative stay. 2-field lymphadenectomy using the Ivor Lewis procedure or trans-hiatal esophagectomy is more commonly performed for esophageal adenocarcinoma in Western countries [5]. Because differences in oncological features and health insurance systems may result in differences in surgical procedures and postoperative stay, it remains unclear whether the predictive factors are applicable to assess patients in Western countries [7, 53]. Fourth, delayed emptying of the gastric conduit was eliminated because there were no patients with endoscopic pyloric dilation and surgical intervention [54, 55]. Despite these limitations, this study is the first to address major complications related to gastric conduit after TE.

## Conclusions

In conclusion, 3FLND and the levels of CPK and CRP at 1POD after TE were predictive factors for major complications related to gastric conduit. Two or more factors in 3FLND and the high levels of CPK and CRP at 1POD after TE were identified as the risk model for major complications related to gastric conduit after TE.

## Abbreviations

3FLND: Three-field lymph node dissection
AUC: The area under the ROC curve
BMI: Body mass index
CI: Confidence interval
CPK: Creatine phosphokinase
CRP: C-reactive protein
IQR: Interquartile ranges
OR: Odds ratio
POD: Postoperative day
ROC: Receiver operating characteristic
SD: Standard deviation
TE: Thoracoscopic esophagectomy
